# Metformin, chronic nephropathy and lactic acidosis: a multi-faceted issue for the nephrologist

**DOI:** 10.1007/s40620-020-00941-8

**Published:** 2020-12-29

**Authors:** Filippo Mariano, Luigi Biancone

**Affiliations:** Department of Medical Sciences, University of Turin, Turin, Italy

**Keywords:** Metformin, Chronic nephropathy, Lactic acidosis, Acute kidney injury

## Abstract

Metformin is currently considered a first-line therapy in type 2 diabetic patients. After issuing warnings for decades about the risks of lactic acidosis in patients with chronic nephropathy, metformin is now being re-evaluated. The most recent evidence from the literature has demonstrated both a low, acceptable risk of lactic acidosis and a series of favorable effects, which go beyond its hypoglycemic activity. Patients treated with metformin show a significant mortality reduction and lower progression towards end-stage renal disease in comparison with those treated with other hypoglycemic drugs. Concerning lactic acidosis, in the last few years it has been shown how lactic acidosis almost always developed when patients kept taking the drug in the face of a concomitant disease or situation such as sepsis, fever, diarrhea, vomiting, which reduced metformin renal clearance. Actually, clearance of metformin is mainly renal, both by glomerular filtration and tubular secretion (apparent clearance 933–1317 ml/min, half-life < 3 h). As regards treatment, in cases of lactic acidosis complicated by acute kidney injury, continuous renal replacement therapy (CRRT) plays a crucial role. Besides the elimination of metformin, CRRT  improves survival by correcting acidosis, electrolyte alterations, and maintaining fluid balance. Lactic acidosis almost always develops because of preventable drug accumulation. Therefore, prevention is a key factor. Patients should be aware that discontinuation for a limited time does not affect their health, even when it may be inappropriate, but it may avoid a serious, potentially fatal adverse event.

## Introduction

In April 2016, the Food and Drug Administration (FDA) stated that the antidiabetic drug metformin could be used “in patients with mild renal failure and, in particular situations, even moderate” [1].

After issuing warnings for decades about the risks of metformin-induced lactic acidosis in patients with chronic kidney disease (CKD), the FDA concluded that the previous guidelines were overly restrictive for metformin. Metformin was approved in 1995, with contraindication in patients with renal disease or dysfunction (serum creatinine levels > = 1.5 mg/dl for males and > = 1.4 mg/dl for females), or in the presence of impaired creatinine clearance.

Although lactic acidosis, the main complication related to the use of metformin, was a serious and potentially fatal condition, during the last 20 years a higher rate of lactic acidosis has not been found in patients taking metformin compared to the general population [2–4]. Furthermore, some investigations have shown that in the real world metformin was used in high percentages in patients below the kidney function threshold values set by the FDA ((creatinine of 1.5 mg/dl for males, and 1.4 mg/dl for females)). Nonetheless, no significant increase in lactic acidosis cases occurred, and when cases did occur in metformin-treated patients, they were almost always associated with some triggering event such as sepsis, or severe hemodynamic failure. Therefore, for CKD diabetic patients suffering from a syndrome rather than a single disease, metformin was simply a risk factor that predisposed to the onset of a severe complication [5–7].

After 2016 the European Medicines Agency (EMA) started reviewing the use of metformin in patients with different levels of renal impairment, aiming at harmonizing the prescription in all European Union countries. After years of consolidated use, metformin has proved to be a safe and efficient drug, with a favorable cost-benefit ratio (as high as not to be comparable) to any other antidiabetic drug.

On the horizon of diabetes therapy, new “players”, such as sodium-glucose co-transporter type 2 inhibitors (SGLT2i) and glucagon-like peptide 1 receptor agonists (GLP1RA) are currently emerging. Even though their real role in the future of diabetic therapy is not fully defined, SGLT2i and GLP1RA have great potential for the prevention of cardiovascular (CV) risk and CKD in diabetic patients [8, 9]. The recent guidelines of the ESC 2019 and the ADA-EASD 2020 Consensus suggested adding these drugs to metformin to reach the HbA1c target, and in high-risk CV patients regardless of the HbA1c values achieved with metformin treatment [8–12].

The intrinsic value of the metformin molecule remains undisputed for patients with CKD as it is always the first-line drug treatment, at least as long as estimated glomerular filtration rate (eGFR) is ≥ 30 ml/min/1.73 m2 [11, 12], even though some concerns remain regarding the reported association with lactic acidosis.

The purpose of this paper is to provide a nephrologist’s point of view on the relationship between metformin, renal function, CKD, and its potential long-term benefit. We will also focus on lactic acidosis requiring renal replacement therapy (RRT), with an update on the modalities of dialysis that are more appropriate for this severe complication.

### Metformin and renal function

Metformin (dimethylbiguanide, MW 129.17 daltons) was first synthesized in 1922, and since 1958 it has been used in the control of hyperglycemia in type II diabetes [13]. Metformin is a biguanide, chemically developed from galegina, a natural guanidine compound found in Galega Officinalis (French lilac).

**Fig. 1:**
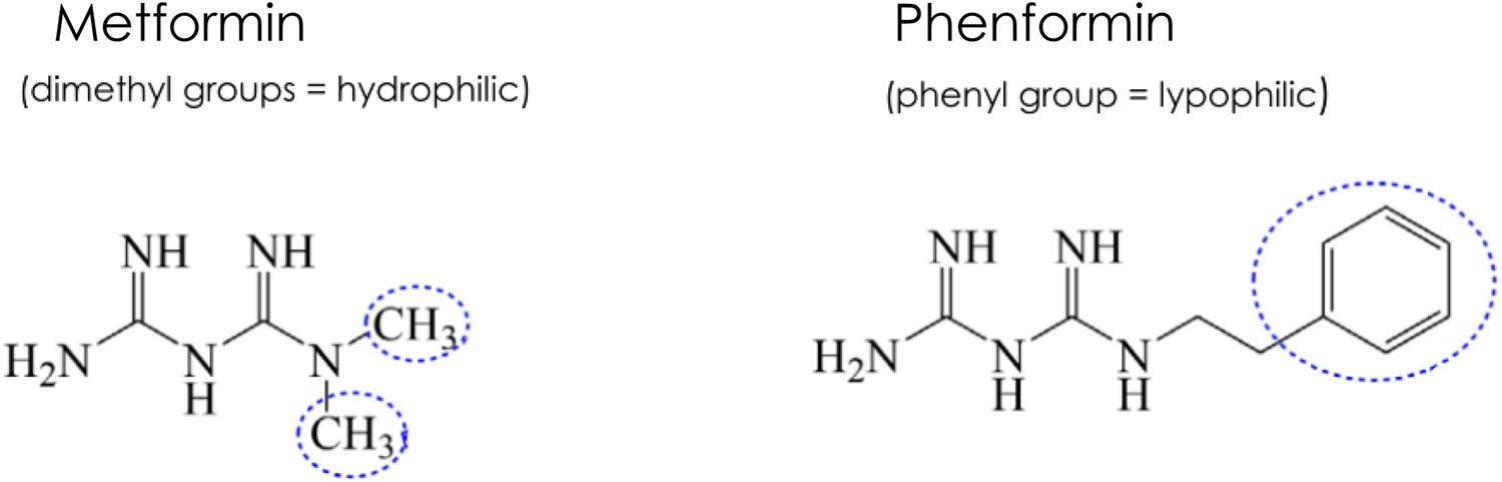
Chemical structure of biguanides metformin and phenformin

Metformin is the natural successor of phenformin, that was used as the reference oral biguanide for many years. Phenformin is a hydrophobic molecule, rapidly absorbed by the intestine, extensively metabolized in the liver to 4-hydroxy-phenformin, and with a long half-life of 11 h [14]. Phenformin was withdrawn from the market in the late 1990 s for inducing fatal lactic acidosis, mainly related to the different ability of the subjects to metabolize the drug in the liver. In subjects with low metabolic capacity, accumulation of the drug and toxic levels occurred leading to potential fatal lactic acidosis [15, 16]. As shown in Fig. 1, metformin has 2 methyl groups instead of a phenyl group of phenformin [17]. At physiological pH, metformin is a cationic hydrophilic molecule (> 99.8%). Despite its low molecular weight, its chemical characteristics strongly limit passive diffusion in cell membranes. Furthermore, unlike phenformin, metformin does not undergo metabolism and it is eliminated unchanged [13].

Erythrocytes can slowly uptake metformin. Six hours after a single dose, when plasma concentrations are negligible, metformin concentrations in erythrocytes exceed those in plasma. Erythrocytes work like a separate compartment, which slowly keeps releasing the accumulated drug. However, this prolonged elimination by the erythrocyte compartment challenges the traditional view that the drug could be shortly cleared even after short dialysis therapy. In the case of lactic acidosis due to accumulation of metformin, the drug is detected in plasma and erythrocytes for up to 13 days [18].

Metformin is administered *per os* 2–3 times a day in a cumulative dose that rarely exceeds 3 g/day. Metformin is absorbed in the upper part of the small intestine (duodenum, jejunum) through specific membrane transporters. Gastrointestinal absorption is incomplete, with a discrete intra-subject as well as inter-subject variability. The mean bioavailability (F) is at about 55% [13, 19].

Metformin plasma protein binding is negligible. In healthy and/or diabetic patients with normal renal function metformin half-life is 3–5 h depending on the oral formulation (immediate or extended-release) (see Table 1).

**Table 1:** Pharmacology of metformin in normal subjects and in chronic renal failure (adapted from 13,18–20)

Parameter	Normal subjects	Chronic renal failure
Bioavailability (%)	50–60	50–60
Distribution volume (L)^a^	500–600	–
Half-life (hours)^b^	3.0	4.5–13.0
Clear/F (ml/min)^b^	933–1317	200–850
Toxic threshold plasma level (mg/L)^c^	5.0	5.0
Threshold-dose (p.o mg/day)^d^ BCrC 120 ml/min	3000	–
BCrC 60 ml/min	–	2000
BCrC 30 ml/min	–	1000
BCrC 15 ml/min	–	500

^a^Values are referred to patients with type 2 diabetes mellitus with normal renal function

^b^The values are referred for oral IR formulations and to the different stage of renal failure for chronic renal failure (Ref [19, 20])

^c^Plasma peak levels for XR formulation and as equal split doses for IR formulation

^d^Recommended daily doses for XR formulation and as equal split doses for IR formulation. Doses in chronic renal failure are according to renal function (Ref 20)

*Clear/F* clearance after oral administered dose, *BCrC* blood creatinine clearance, *IR* immediate release, *XR* extended release

The elimination of the drug takes place in the urine, by tubular secretion, and as an unchanged drug. After oral administration, the mean renal clearance and apparent total clearance (Clear/F) are estimated to be 510 ± 130 ml/min and 1140 ± 330 ml/min, respectively. These values, which are 4.3 and 10.7 times the creatinine clearance, underline the role of the kidney in metformin elimination. Since metformin clearance always maintains a direct proportion to creatinine clearance for the different values of renal function [20], with an increasing degree of renal failure the dosage should be proportionally decreased (Table 1).

Intestinal absorption, liver uptake, and renal excretion are largely mediated by the specific transport system Organic Cation Transporter (OCTs) and Multidrug And Toxin Extrusion Transporter (MATE) [19]. Specific variants of these transporters (OCT1, polymorphism rs622342) are known to have been associated with a decreased hypoglycemic effect in heterozygotes, and with a missing effect in homozygotes. The specific transporter variants can significantly change absorption, hepatic uptake, and renal secretion, and therefore they can modulate the pharmacokinetics/pharmacodynamics of metformin in the individual subject [21].

### Metformin prescription

Current guidelines state that in normal subjects (eGFR > 90 ml/min) the usual starting dose varies between 500 mg and 850 mg of metformin 2 or 3 times a day during or after meals, with a maximum recommended daily dose of 3 g. In patients with renal impairment, eGFR must be evaluated before starting treatment, and subsequently at least once a year, or at least every 3–6 months for patients with eGFR 30–59 ml/min (11,12). Regarding the dose, in patients with eGFR between 60 and 89 (stage II KDIGO) the maximum dose should be less than 3 gr/day in 2–3 administrations, in patients with eGFR between 45 and 59 ml/min the dose must be less than 2 gr/day, and between 30 and 44 ml/min less than 1 gr/day.

Metformin is contraindicated in patients with eGFR less than 30 mL/min [11, 12, 22]. In patients with eGFR between 30 and 60 mL/min, the known factors increasing the risk of lactic acidosis (other drugs, serious respiratory and circulatory comorbidities, septic state, liver failure) must be examined before considering the start of metformin treatment. Furthermore, the starting dose should not exceed half the maximum indicated dose [11, 12, 22].

### Metformin and CKD

The use of metformin in CKD has always been controversial and strongly limited in the last decades by the potential cases of associated lactic acidosis [6, 11, 12, 23].

However, for patients with CKD recent evidence has demonstrated on one hand a low risk of lactic acidosis [23], and on the other a series of favorable effects that go beyond hypoglycemic activity, including the possibility of slowing down the decline of renal function [3, 4, 24–28].

In 2010, for the first time, a metformin protective effect for patients with chronic renal failure was described in a cohort of 19,691 patients with type II diabetes and documented atherosclerotic disease. In the subgroup of 5031 patients with KDIGO stage III nephropathy (eGFR 30–60 ml/min) mortality rate was significantly lower in metformin-treated patients than in non-metformin-treated patients. This effect was more evident in patients with chronic KDIGO stage III-B (eGFR 30-45 ml/min) [3].

In 2012, a second large study confirmed the positive relationship between metformin and mortality in CKD patients. A national study, carried out in Sweden on 51,675 diabetic patients, showed that in patients with CKD KDIGO stage III-A (eGFR 45-60 ml/min) the use of metformin in monotherapy for a median follow-up of 3.9 years was associated with significant cardiovascular protection and reduced mortality without any significant increase in lactic acidosis rate [4]. In 2017, a meta-analysis of 5 large studies on metformin use among adults with type 2 diabetes and comorbid moderate-to-severe CKD (eGFR < 45 mL/min/1.73m2 or even 30 mL/min), chronic heart failure, or chronic liver disease with impaired hepatic function demonstrated that the relative chance of dying during follow-up was 22% lower for patients taking metformin than for those not taking it (HR 0.78; 95% CI 0.63–0.96) [25].

Other reports focusing on patients with CKD confirmed a significant reduction in major cardiovascular events (MACE) in metformin-treated subjects compared to sulphonylurea-treated ones [26–28]. As to the severity of impaired renal function, metformin should not be used in patients with eGFR < 30 ml/min (stage 5 KDIGO) because of an observed significant increased mortality. Conversely, metformin was safe when used in patients with eGFR > 30 ml/min/1.73 m2 [29, 30]. As to the risk of acidosis, in a large cohort of patients with eGFR of at least 30 mL/min/1.73 m2, metformin was not associated with incident hospitalization with acidosis, even after accounting for a change in eGFR stage over time. In comparison with metformin nonuse, there was a higher acidosis risk associated with use only at eGFR less than 30 mL/min/1.73 m2 (adjusted HR, 2.07; 95% CI, 1.33–3.22) [31].

Interestingly, after the FDA changed the labeling regarding metformin contraindications for diabetes patients with CKD, by eGFR and not by creatinine values, a reduction in racial and sex disparities of metformin prescription was observed. Before 2016, Black patients with eGFR of 30–44 ml/min/1.73 m2 were prescribed metformin less often than their White counterparts, whereas this phenomenon was significantly attenuated after the FDA label change (aPR, 0.90; 95% CI, 0.74–1.09; P value for interaction by period = 0.04) [32].

A recent retrospective, observational paper studied the primary outcomes (mortality and progression of chronic kidney disease) and the secondary outcome (incidence of lactic acidosis) of 10,862 type 2 diabetic patients with CKD for a long average follow-up of 7.3 ± 4.8 years. Mortality was significantly reduced for 4597 “metformin-users” compared to 6265 “no metformin-users”. In addition, the study demonstrated significantly reduced progression towards end-stage renal disease (ESRD) assessed as renal replacement treatment need (aHR 0.66; 95% CI 0.59–0.76; *P* = 0.001) [33]. The significantly slower decline of renal function in “metformin-users” vs “no-metformin users” was also present after data analysis with “Propensity Score Matching”, and with Kaplan–Meier curves constructed with patient stratification according to KDIGO stage 3 and stage 4. In particular, the benefit on mortality and progression of nephropathy was most evident for patients with eGFR between 30 and 44 ml/min [33]. As regards safety, only one case of lactic acidosis associated with metformin use was demonstrated (aHR 0.92; 95% CI 0.668–1.276; *P* = 0.629) [33].

The metformin protective effects described above were not seen in the TREAT study, which failed to demonstrate the statistical significance of a renal protective effect (aHR 1.01; 95% CI 0.65–1.55; *P* = 0.98) [30]. It has been hypothesized that this result discrepancy was due to the short follow-up of the TREAT study (average follow-up: 2.5 years, with a maximum of 4.5 years) [30]. To highlight a significant risk reduction on the progression towards ESRD, metformin treatment is required for more than 2.6 years, and more than 4.5 years when considering 95% CI [33].

Several experimental data support the observed metformin-induced renal protection. Metformin exerts its metabolic cellular effects essentially through an activated AMP-protein kinase (AMPK) pathway [34]. The AMPK pathway is important not only for increased peripheral glucose uptake and (in the path of) gluconeogenesis (both related to insulin signal), but also for the mechanisms of cell stress protection. In tubular cells either a hyperglycemic state or proteinuria down-regulates defensive mechanisms, such as the AMPK pathway and autophagocytosis, and up-regulates pathological pathways such as those of epithelial-to-mesenchymal transition, oxidative stress, hypoxia, and apoptosis [35]. Metformin has the intrinsic potential of reducing tubulo-interstitial fibrosis, the epithelial-mesenchymal transition, and the expression of TGF-1beta, a well-known fibrogenic factor [35, 36].

### Metformin, lactic acidosis and AKI

Metformin-associated lactic acidosis (MALA) is defined by a blood lactate level ≥ 5 mmol/L, decreased pH and bicarbonates, and an increased anion gap. The therapeutic range of metformin is 2–4 mg/L. In MALA patients, metformin blood levels are generally high, largely exceeding 5 mg/L, and the therapeutic range of 2-4 mg/L [37, 38]. Metformin at a high dose exerts per se toxic effects. In healthy patients during (anti-preservative attempts) with metformin at high doses, the high blood concentrations of metformin exceeding 50 mg/L induced a state of severe lactic acidosis (lactate levels > 25 mmol/L) burdened by a high mortality rate [37, 38].

The lactate increase means an increased lactate/pyruvate ratio. Generally speaking, the mechanisms underlying lactate increase and biguanide intoxication involve several metabolic pathways [34]. Metformin can inhibit the mitochondrial respiratory chain (complex 1) and mitochondrial glycerophosphate dehydrogenase, and therefore direct the cell towards an anaerobic respiratory shift [34, 39]. Accumulated pyruvate upstream from Krebs cycle is converted to lactate by lactic dehydrogenase. Furthermore, lactate cannot be efficiently consumed in liver gluconeogenesis because of inhibition of the pathway by metformin in an oxide-reduction-dependent manner [39]. Therefore, lactate increase is sustained both by excess production and reduced consumption in the liver.

However, in clinical settings the real role of metformin in MALA onset is still not well defined. As a matter of fact, it has been shown that the incidence of lactic acidosis for metformin users was not substantially different from that observed in patients treated with other oral hypoglycemic agents [23], and risk factors such as the presence of renal failure, cardiovascular disease and age > 65 years, were crucial for the onset of lactic acidosis [23]. Furthermore, even if metformin did not increase the risk of acute kidney injury (AKI) [40], AKI is still a clinical condition that is frequently described in association with MALA [41–45], and the decline of renal function paralleled the accumulation of metfomin at plasma levels capable of inducing lactic acidosis [41, 42]. In addition, in AKI patients MALA is worsened as the kidney fails the function of a lactate-consuming organ.

### Metformin, lactic acidosis and RRT

Data concerning the incidence of severe MALA cases requiring RRT are scarce, often incomplete, and not so conclusive. A recent survey in north-west Italy focused on MALA cases admitted to the ICU and needing renal replacement therapy for AKI [45]. The source of data was the network of Nephrology and Dialysis Centers of Piedmont-Aosta Valley regions, where all ICU replacement treatments involved the nephrology community [46].

**Fig. 2:**
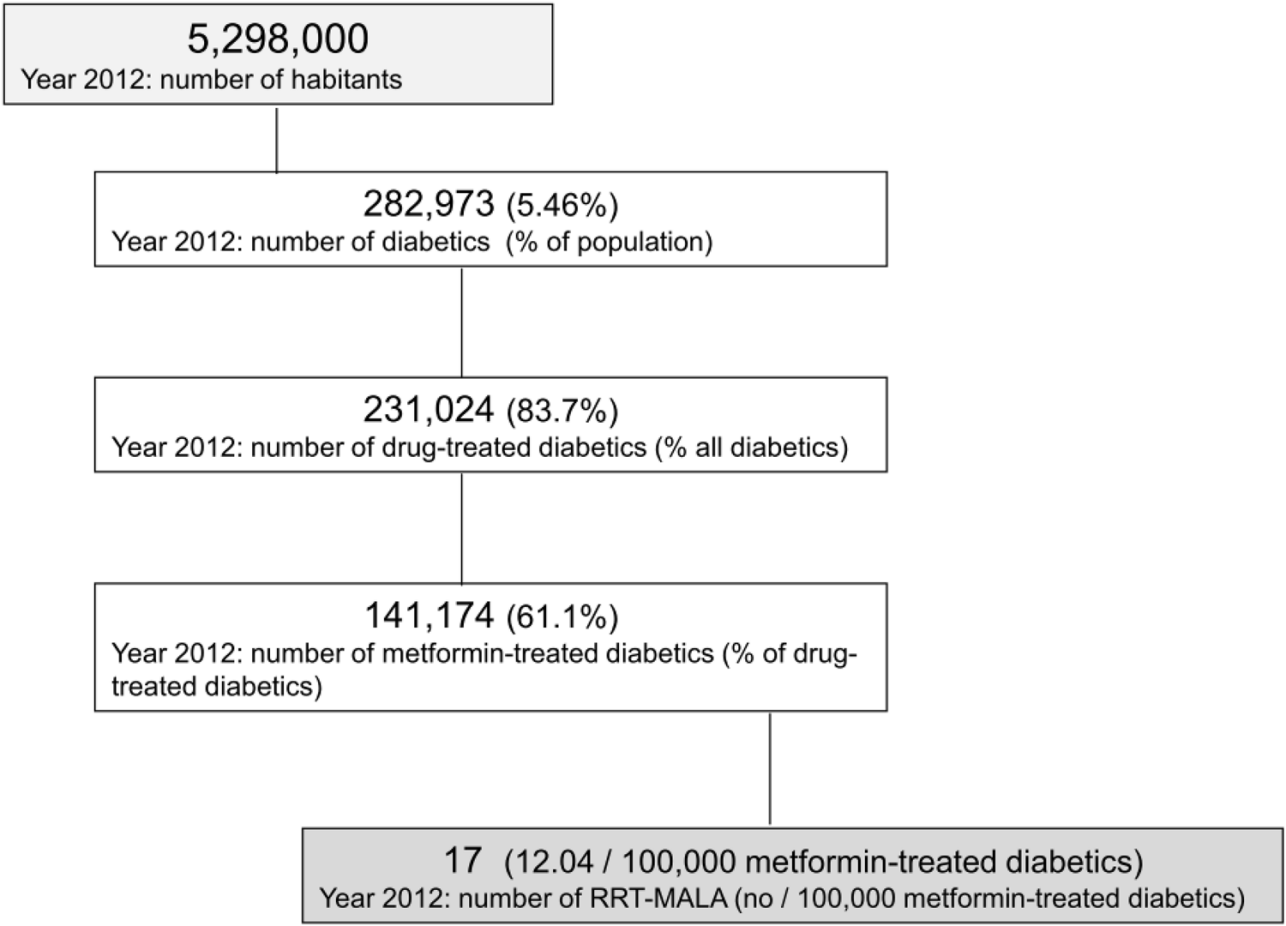
Flow chart of RRT-MALA cases recorded in Piedmont and Aosta Valley regions in 2012 (modified from Ref 45). RRT-MALA: cases of metformin-associated lactic acidosis treated with renal replacement therapy

In 2012, 17 patients suffering from a serious form of MALA were admitted to the ICU of Piedmont-Aosta Valley regions requiring renal replacement therapy (MALA-RRT patients) (Fig. 2). According to the Diabetes Registry [47], 289,970 inhabitants (5.46%) out of 5,298,000 were affected by diabetes, and 231,024 (4.36% of the population) were treated with drugs. Of these 231,024 diabetics, 141,174 patients (61.1%) were on metformin therapy. Therefore, in 2012 the incidence of MALA-RRT patients in north-west Italy was 12.04 cases/100,000 metformin-treated patients (Fig. 2).

Over a 6-year period from 2010 to 2015, 117 cases of MALA-RRT occurred, with a survival rate of 78.3% (92/117) (Table 2). At admission, all MALA-RRT patients had a picture of AKI stage III, and in 35% of cases AKI was superimposed on CKD. Most of the patients (> 70%) were shocked and required cardio-circulatory support with vasoactive amines, and septic shock was diagnosed in 25% of patients. Oliguria or anuria was present in 90% of patients, and dehydrationand/or gastrointestinal losses were observed in 80% of patients. Surprisingly, the metformin mean dose prescribed in MALA-RRT patients was relatively low (1.6 g/day) [45]. Gastrointestinal losses and dehydration status likely played an important role in the accumulation of metformin driven by impaired renal clearance.

**Table 2:** Demographic data and RRT parameters of 117 metformin-associated lactic acidosis patients admitted in ICUs and treated with renal replacement therapy (modified from Ref. [45])

Patients no. (F/M)	76/41 (64.9%)
ICU survival (survived/dead, %)	92/25 (78.6%)
Age (years, mean)	71.6
*At RRT start*	
Creatinine (umol/L, mean)	598.8
pH (arterial blood, mean)	7.04
Lactate (mmol/L, mean)	12
Time interval before RRT start (hours, mean)	3.2
*RRT clinical parameters*	
Duration of RRT (days, mean)	3.9
Dialysis dose (ml/Kg/day, mean)	977.7
Predilution (% of infusion, mean)	31.8
Heparin anticoagulant	84/19 (81.5%)
HF or HDF techniques (yes/no (%))	91/18 (83.4%)
CRRT or PIRRT modalities (yes/no (%))	87/22 (79.8%)

*RRT* renal replacement therapy, *HF* hemofiltration, *HDF* hemodiafiltration, *CRRT* continuous renal replacement therapy, *PIRRT* prolonged intermittent renal replacement therapy

In 2020, a French observational study [48] involving 133 patients reported similar data. Sepsis was reported in 77/133 patients (58%), a shock condition in 64/133 patients (48%), and digestive disorders in 87/133 patients [48].

### Current evidences for extracorporeal treatment of MALA-RRT patients

Current guidelines for the use of RRT in MALA patients state that RRT is recommended in case of lactic acid concentration > 20 mmol/L, pH ≤ 7.0, shock, failure of standard supportive measures, or decreased level of consciousness [49].

Metformin is a small hydrophilic molecule (MW 165 D) with negligible protein binding (< 1.1%). The diffusive technique at high blood flow allows high metformin clearance from plasma up to 170 ml/min, and due to its high clearance rate hemodialysis was considered the first-line therapy for MALA patients [49].

However, over the years convective or mixed techniques in continuous or prolonged modalities have shown a high survival rate of MALA patients, despite being applied at a fluid exchange rate much lower than classical hemodialysis (41,45,48,50,51). Keller et al (50) treated 6 patients (3 continuous venovenous hemodiafiltration [CVVH], 3 continuous venovenous hemodialysis [CVVHD]) by continuous renal replacement therapy (CRRT) with a favorable outcome in all cases. They demonstrated a significant improvement in metabolic acidosis and reduction of plasma metformin concentrations within the first 24 h, and normalization on the second day. CRRT was carried out early, at a mean effluent flow rate of 34 ± 6 ml/kg/h. A protective effect of RRT was suggested by Peters et al. [41] who found that the 30% mortality rate was similar for 16 patients treated with intermittent hemodialysis and for 14 non-dialyzed subjects, despite greater illness severity in the former group.

Most cases of MALA admitted to ICUs are subjects with typical chronic metformin toxicity associated with volume depletion, and/or AKI with oligo-anuria. Taking into account the high volume of distribution of metformin (up to 600 L) and the time-dependent partitioning of metformin over time into the intracellular compartment, extracellular compartment, red blood cells, and plasma, metformin becomes less and less available for removal when hemodialysis at high blood flow rate (QB) is used. In a recent report on MALA related to massive ingestion of metformin (plasma metformin peak concentration of 40.7 ug/L) and AKI treated by hemodialysis for 6 h, the mean whole blood and plasma clearances of metformin were 37.74 and 47.27 mL/min (QB 250 ml/min, dialysate flow rate [QD] 600 ml/min, high-flux polysulfone filter 1.8 m^2^, metformin sieving coefficient of 0.15) [52]. During hemodialysis urine output recovered, and mean urine clearance was 21.88 mL/min. The amount of removed metformin was 888 mg by 6 h of hemodialysis, and 142 mg in the urine during this time [52]. Thus, 6 h of hemodialysis was poorly efficient as it removed only a « tablet » of metformin.

Conversely, the amount of metformin removed by continuous treatment can be more efficient. Continuous treatment works at a lower effluent rate of 40–50 ml/min, but for a long enough time to allow the drug to balance between the body compartments. Furthermore, clinical experience shows that when the CRRT improves hemodynamic instability and acid–base status, lactate concentration usually decreases and urine output recovers. Therefore, besides the elimination of metformin CRRT plays a crucial supportive role for the initial survival of the patients by correcting acidosis, electrolyte alterations, and maintaining fluid balance.

In the experience of 117 RRT-MALA patients treated in ICUs, at the start of RRT mean plasma creatinine value was 598 umol/L, and mean pH and lactate levels were 7.04 and 12 mmol/L, respectively. RRT started early, at a mean of 3.2 h after admission, and lasted a mean of 3.9 days (Table 2). The mortality rate was 21.4%. Continuous or prolonged modalities or RRT, as well as pure convective or mixed treatments, were applied in more than 80% of patients at a mean dialysis dose of 977 ml/Kg/day (Table 2).

Even if the best modality of RRT for MALA patients is not clearly defined [49], it is conceivable that the ideal extracorporeal treatment should be early and continuous (or at least prolonged), and should last until a sustained correction of acid–base status and hemodynamic stability are obtained (Table 3). Current guidelines suggest that cessation of extracorporeal treatment is indicated when lactate concentration is less than 3 mmol/L and pH is more than 7.35 [49]. The rate of exchange with a bicarbonate-containing solution should be more than the standard recommended dose of 25 ml/Kg/hour, modulated to correct acid–base status and electrolyte alterations. As to dialysis modalities, CRRT should be preferentially carried out with high permeability hemodialyzers, either by diffusion, convection or mixed techniques (Table 3).

**Table 3:** Suggested RRT prescriptions for patients with metformin associated lactic acidosis

	Indications	References
Start of RRT	Lactic acid concentration > 20 mmol/L pH ≤ 7.0, shock, failure of standard supportive measures or decreased level of consciousness	[41, 49]
Timing of RRT	Early as soon as possible	[45, 50]
Duration of RRT	Continuous, or at least prolonged	[41, 45, 48, 50–52]
Dialysis dose (ml/Kg/day)	> 25 ml/Kg/hour (35–50??)	[45, 50]
RRT with bicarbonate solution	Yes, > 32 mmol/L	[41, 45, 49]
RRT modality	Diffusive, convective or mixed	[41, 45, 48–52]
Cessation of RRT	When lactate concentration is < 3 mmol/L and pH is > 7.35	[49]

*RRT* renal replacement therapy

## Conclusions

Metformin is a well-known drug that has been used for more than 70 years, and that is still in the headlines due to its surprising pleiotropic effects. Inserted by the WHO in the list of the safest and most effective drugs named “Essential Medicines”, metformin is the most widely used oral antidiabetic drug in the world. Nowadays, metformin is a generic drug with a highly favorable cost-benefit ratio (monthly cost of therapy: from 2 to 6 euros), whose use is crucial for diabetes care in developing countries.

The clinical experience accumulated in these years has shown how lactic acidosis almost always develops because of a preventable drug accumulation. Patients taking the drug in the face of a concomitant complication which has led to reduced renal clearance of metformin (fever, diarrhea, vomiting, general malaise) must discontinue metformin. In the treatment of MALA patients, when standard supportive measures fail extracorporeal treatment should always be considered as early adjunctive therapy.

In conclusion, prevention is a key factor in reducing cases of MALA. By providing basic capillary information, patients should be made aware of the fact that even inappropriate discontinuation for a limited time does not affect their health, but it may prevent serious, potentially fatal adverse events.
